# Impact of Benzodiazepine Delorazepam on Growth and Behaviour of *Artemia salina* Nauplii

**DOI:** 10.3390/biology13100808

**Published:** 2024-10-10

**Authors:** Chiara Fogliano, Rosa Carotenuto, Claudio Agnisola, Chiara Maria Motta, Bice Avallone

**Affiliations:** Department of Biology, University of Naples “Federico II”, 80126 Naples, Italy; chiara.fogliano@unina.it (C.F.); rosa.carotenuto@unina.it (R.C.); agnisola@unina.it (C.A.); bice.avallone@unina.it (B.A.)

**Keywords:** psychotropic drug, developmental toxicity, heart rate, locomotory test, growth rate, eye development, lipid reserve consumption

## Abstract

**Simple Summary:**

Benzodiazepines are drugs resistant to sewage treatment, persisting in aquatic environments. Their presence notably increased after the COVID pandemic due to the anxiety induced by health risks and lockdown. Prior studies have shown that benzodiazepines adversely affect both the larval and adult stages of various species, impacting behaviour and embryonic development. This study focused on delorazepam’s effects on the naupliar stages of *Artemia salina*, a small saltwater crustacean. The delorazepam treatments (1, 5, and 10 µg/L) increased hatching rates and caused growth desynchronisation. Treatment altered lipid reserve consumption, with lipid globules persisting in the advanced naupliar stages. Locomotory activity significantly decreased at the highest concentration (10 µg/L). Although no teratogenic effects were observed, minor damage was noted in the posterior trunk and eyes, indicating environmental toxicity targets. This study highlights the urgent need for further research and monitoring of benzodiazepines as aquatic contaminants, emphasising their inclusion in ecological risk assessments.

**Abstract:**

Benzodiazepines, a significant group of newly recognised water contaminants, are psychotropic medications prescribed for common anxiety symptoms and sleep disorders. They resist efficient degradation during sewage treatment and endure in aquatic environments. Their presence in aquatic matrices is increasing, particularly after the recent pandemic period, which has led many people to systematically use benzodiazepines to manage anxiety. In previous studies, an important interference of this class of drugs on both the larval and adult stages of some aquatic species has been demonstrated, with effects on behaviour and embryonic development. This study examined the influence of delorazepam, a diazepam metabolite, on *Artemia salina* development to gain insight into responses in naupliar larvae. Results demonstrated that treatments (1, 5, and 10 µg/L) increase the hatching percentage and induce a desynchronisation in growth. Mortality was only slightly increased (close to 10% at six days post-hatching), but lipid reserve consumption was modified, with the persistence of lipid globules at the advanced naupliar stages. Locomotory activity significantly decreased only at 10 µg/L treatment. No teratogenic effects were observed, though modest damages were noticed in the posterior trunk and eyes, two targets of environmental toxicity. The negative impact of delorazepam on *Artemia salina* adds to those already reported in other species of invertebrates and vertebrates, which are not yet considered targets of these drugs. This study underscores the need for further research and immediate attention to this class of contaminants and the importance of monitoring their presence during environmental risk assessments.

## 1. Introduction

In recent decades, technological advances in water pollution assessments have allowed the identification of new environmental contaminants. Among these, pharmaceuticals are raising growing concern since they are discarded in wastewater directly and as excreted metabolites [1,2,3]. Psychoactive compounds, and benzodiazepines (BZDs) in particular, are among the most used and abused drugs [4,5]. Consequently, they may reach concentrations that can affect non-target species by activating the same receptor-based mechanisms activated in the patients for which the drugs were designed [6,7]. BZDs contaminate all the environmental matrices, with the highest concentrations reported along the coasts, especially in the proximity of large cities and estuaries [2,8,9,10,11,12,13]. A recent increase occurred, coinciding with the COVID-19 pandemic [14].

Therefore, BZD monitoring deserves particular attention: these drugs are effective at the lowest environmental concentrations [15,16], and they may affect both invertebrates and vertebrates since GABA-A and TSPO receptors are highly conserved [17,18]. Indeed, not only behavioural alterations [19,20,21,22,23,24] but also cytological, physiological, and biochemical interferences [19,20,21,22,25] may be induced in both adults [20,22] and embryos/larvae [19,26].

To expand knowledge of the harmful effects of BZDs on non-target organisms, the present work aimed to evaluate the impact of delorazepam (DLZ), a metabolite of diazepam [27], on the early developmental stages of the brine shrimp *Artemia salina*. Nauplii are excellent bioindicators in evaluating water quality and can provide information on the responses of zooplanktonic species to environmental perturbations [28]. *Artemia salina* nauplii are also good models in toxicity studies: they have been used to determine the effects of heavy metals [29], microplastics [30], pesticides [31], and biocides [32], to cite a few examples. DLZ was tested at 1, 5, and 10 µg/L, the dosages that proved teratogenic for embryos of the African clawed frog *Xenopus laevis* and stony sea urchin *Paracentrotus lividus* [19,26] and to interfere with the behaviour of the great ramshorn snail *Planorbarius corneus* [22].

To evaluate the possible interference with *Artemia salina* development, conventional endpoints were analysed: the hatching percentage, mortality, and growth rate. In addition, considering the phenotypical abnormalities induced in *X. laevis* and *P. lividus* [19,26], *A. salina* nauplii were examined for anomalies in body axes, gut, eye, and appendages development. Particular attention was directed to lipid reserve consumption, as anomalies were already observed in *X. laevis* [21]. The drug’s sedative effects were also considered, and the heartbeat rate was determined in nauplii at stages L4 and L5. In addition, locomotory performances were tested at the lowest and highest concentrations (1 µg/L and 10 µg/L), expected to be the least and most effective. Antennal stroke frequency and mean velocity were determined on nauplii exposed either from hydration (since embryogenesis) or, for comparison, from hatching.

## 2. Materials and Methods

### 2.1. DLZ Solution Preparation

For the treatment, a widely consumed pharmaceutical product obtained from a commercial source was used; in the form of oral drops, it contains the active principle DLZ dissolved at a 1 mg/mL concentration in water and excipients (in unspecified quantities). Solutions were prepared by diluting the product in artificial seawater to reach three final concentrations: 1 μg/L, considered to be an environmentally realistic concentration [33], and two higher concentrations, 5 and 10 μg/L, for comparison, and in consideration of the increasing trend of BZDs’ use and abuse [14]. All concentrations were much lower than the 4–10 mg/L range reported as LC50 for diazepam in crustaceans [34,35]. Controls were grown in pure artificial seawater.

### 2.2. Artemia salina Care and Maintenance

Dried *Artemia salina* cysts (granted hatching percentage > 90%) were incubated at 22 ± 1 °C in artificial seawater (Instant Ocean Sea Salt, Blacksburg, VA, USA, salinity 36 ± 1‰ g/L) under constant aeration and a 16 h light photoperiod (1500 lux). Nauplii were fed twice daily with a commercial algae product specific for *Artemia* and yeast [36]. The experiments were discarded if the mortality percentage in control samples exceeded 10% at two days [37]. Whenever necessary, the nauplii were collected using a wide-mouthed plastic pipette to avoid damaging them, using a light source [38].

### 2.3. Hatching Test

A total of 80–100 dried cysts were placed in 3 mL wells of a 24-multiwell plate; four wells were set for each treatment, and tests were repeated in triplicate (n = 1108 ± 54.3 cysts/treatment). The plates were incubated at 22 ± 1 °C, with a 16 h light photoperiod for 48 h. The nauplii (instar stage I) were collected, transferred into Falcon tubes containing 4% formalin to stop development, and conserved in the fridge at 8 °C. During the examination, aliquots were taken from each sample and observed under a stereomicroscope to count the numbers of nauplii and cysts. Mortality was calculated by applying the formula: dead nauplii/total number of nauplii examined ×100.

### 2.4. Mortality, Larval Staging, and Growth Evaluation

A total of 200 mg of dried cysts was transferred into glass tanks containing 400 mL of pure seawater (control) or seawater containing DLZ at one of the three concentrations. Sampling was performed on days 2, 4, and 6 post-hatching (dph) by picking 8 mL of suspension using a plastic Pasteur pipette. Aliquots were promptly placed under a stereomicroscope to count the number of dead and poorly motile nauplii, those not swimming within a 10 s observation period [39]. The ratio × 100 gave the mortality percentage.

In parallel, nauplii were fixed in 4% formaldehyde and conserved in the fridge at 8 °C. Developmental stages were determined under the microscope, according to Copf et al. [40] and using the body length (stages L1–L3), the thoracopod development (stages L4–L8), and the appearance of paired eyes (L5 stage) as stage reference (Figure S1 for representative pictures of nauplii at different stages). The nauplii at different stages were counted, and data were expressed as percentages of the total number of nauplii observed (not lower than 600 per treatment). All treatments were repeated in triplicate.

### 2.5. Phenotypic Analyses

Fixed nauplii were observed under a light microscope to determine the presence of abnormalities in the body axes, thoracopods, and eyes. The latter two are proven toxicity targets in *Artemia salina* [41,42]. The naupliar length was determined on digital photos of randomly chosen control and treated nauplii (n = 75 per treatment). Data were obtained using the software ImageJ 1.8.0 (last update 22 May 2023).

The presence of lipid reserve was also checked in toto, under the microscope; yolk and lipid globules were put in evidence in transparent L3/L4 nauplii by incident illumination. Their presence was quantified by measuring the absorbance on digital photos [22]. Briefly, images of early L4 nauplii (n = 40 per treatment) were transformed into high-resolution (400 dpi) 8-bit, 256 grayscale images saved in TIFF format. Using ImageJ, density was determined in two identical areas, selected in correspondence with the anterior margin of the first and the posterior margin of the last (fifth) thoracopod buds.

### 2.6. Cardiac Beats Measurement

The heart rate was assessed by non-invasive video analysis of nauplii at stages L4 (n = 40 per treatment/stage) and L5 (n = 40 per treatment). Videos were examined to count the number of beats per 30 s; triplicate measurements were carried out at 30 s intervals.

### 2.7. Determination of Locomotory Performance

DLZ concentrations of 1 μg/L and 10 μg/L were tested to evaluate the effect of DLZ on motility performance. Two protocols were followed: nauplii were exposed at 22 ± 1 °C to DLZ since hydration (protocol A) or after hatching (protocol B). In this latter case, two sub-protocols were adopted: (B1) DLZ was added 24 h post-hatching (1 dph), and (B2) DLZ was added at 2 dph. In protocol B, neonate nauplii were collected and transferred into flasks containing 200 mL of seawater, control, or with DLZ.

Locomotory performance was determined 48 h after the beginning of the exposure. Five batches of nauplii were used for each condition, giving a total of 15 batches for protocol A and 60 batches for protocol B. Ten individuals were analysed from each batch as described above. Mean values of locomotor parameters from these ten individuals from a batch of cysts represented the unit value for a specific dph and treatment. This protocol is resumed in Table S1.

### 2.8. Video Tracking and Evaluation of Locomotory Performance

A squared homemade chamber (1 × 1 cm), built on a microscope slide, was used to record the mobility of individual nauplii. Videos (1920 × 1080 px, 30 fps) were taken with a smartphone video camera from a dissecting microscope using an ocular adapter. The videos were analysed using the Tracker software (Version 6.0.6, 2021) for tracking animals and obtaining the animal mean velocity (BL s^−1^) and beat frequency of antennae (Hz). Further information is reported in the Supplementary Materials (Table S1 and Figures S2–S4).

### 2.9. Statistical Analysis

Data distribution was tested for normality using the Kruskal–Wallis test. Differences in hatching percentage, naupliar length, growth, and mortality were tested with Two-Way ANOVA followed by Tukey’s pairwise comparison test. Values were reported in graphs as a percent of controls, calculated by applying the following formula: mean value of treatment sample/mean value of control sample × 100 [26]. Cohen’s d-test was used to determine the effect size of treatments [43]. Mean values of locomotor parameters from 10 individuals from a batch of cysts represented the unit value for a specific dph and treatment. Data are displayed as mean ± SD of 43–105 batches, depending on the protocol used. Comparisons between treatments were performed using Two-Way ANOVA and the Dunnet post hoc test. Data were analysed using GraphPad Prism version 9 (GraphPad Software, Boston, MA, USA). For all tests, the minimum significance level accepted was *p* < 0.05.

## 3. Results

### 3.1. Effect of DLZ on Hatching and Growth

**Figure 1:**
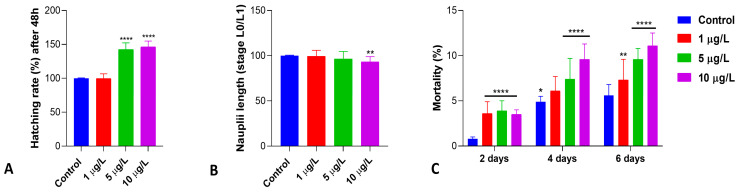
Hatching percentage (**A**), naupliar length (**B**), and mortality (**C**) in *Artemia salina* nauplii exposed to DLZ since hydration for 48 h (**A**,**B**) or up to 6 days (**C**). Two-Way ANOVA followed by Tukey’s pairwise comparison test; * *p* < 0.05; ** *p* < 0.01; **** *p* < 0.000. Number of animals examined: (**A**), n = 1108 ± 54.3 cysts/treatment; (**B**), n = 75 nauplii/treatment; (**C**), n > 600 nauplii/treatment/day.

Exposure of the cysts to DLZ induced a significant dose-dependent increase in the hatching percentage compared to the control (Figure 1A). At the environmental concentration, the percentage remained at values comparable to that of control samples (about 23% of cysts, assumed as 100% of hatching), but in samples exposed to 5 and 10 µg/L, the percentage rose to 142 and 146% (*p* < 0.0001), with an effect size >5. Hatched nauplii, stages L0 to L1 (Figure S1), were significantly shorter at the higher DLZ concentrations compared to controls and DLZ 1 µg/L (*p* < 0.01; effect size 0.1, 0.6, and 1.5, respectively; Figure 1B). In control samples, mortality remained below 6%, a value registered on day 6 dph (Figure 1C). In treated samples, mortality increased significantly, dose-dependently, compared to the controls (Figure 1C). Maximum values, registered at 6 dph, however, remained close to 10%, the value indicated by Vanhaecke et al. (1981) [37] as acceptable at 2 days of treatment. The effect size at 2 days was <1.6 and >4.3 at 4 and 6 days, respectively.

### 3.2. Effects of DLZ on Naupliar Growth

**Figure 2:**
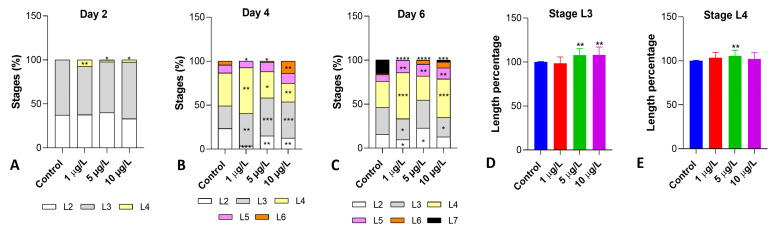
Effects of DLZ on the composition of the naupliar population (**A**–**C**) and naupliar length (**D**,**E**). Significant positive and negative variations are observed in the different samples compared to the relative controls. Two-Way ANOVA followed by Tukey’s pairwise comparison test: * *p* < 0.05; ** *p* < 0.01; *** *p* < 0.001; **** *p* < 0.0001. Number of animals examined: (**A**–**C**), n > 600 nauplii/treatment/day; (**D**,**E**), n = 75 nauplii/treatment/stage.

The analysis of the composition of the naupliar population demonstrated that DLZ interfered with growth. Two dph (Figure 2A), 37 and 63% of the control nauplii were at stages L2 and L3 (Figure S1), respectively. In samples exposed to DLZ, an acceleration was observed as indicated by the appearance of nauplii at the L4 stage (Figure S4) (7% in 1 μg/L and ~2.5% in 5 and 10 μg/L). On day 4 post-hatching (Figure 2B), samples exposed to DLZ 1 and 5 μg/L showed a significant delay demonstrated by the reduced percentage of L6 nauplii (Figure S1) and by the increased percentages of L3 or L4 nauplii. In contrast, in samples exposed to DLZ 10 μg/L, a marked acceleration occurred, as indicated by the significant increase in the percentage of L6 nauplii (14% vs. 4% of controls). On day 6 post-hatching (Figure 2C), all treated samples were delayed: L7 nauplii were well represented in controls (12%) but rare (2.5%) in 10 μg/L samples and completely absent at the two lower DLZ concentrations.

The naupliar length was measured at stage L3 (Figure 2D), at the time nauplii started feeding, and L4 (Figure 2E). Results indicated that significant increases occurred in L3 nauplii exposed to the two higher concentrations (Figure 2D) and in L4 nauplii exposed to 5 μg/L. In all samples, the effect size was <1.5.

### 3.3. Effects of DLZ on Naupliar Anatomy

**Figure 3:**
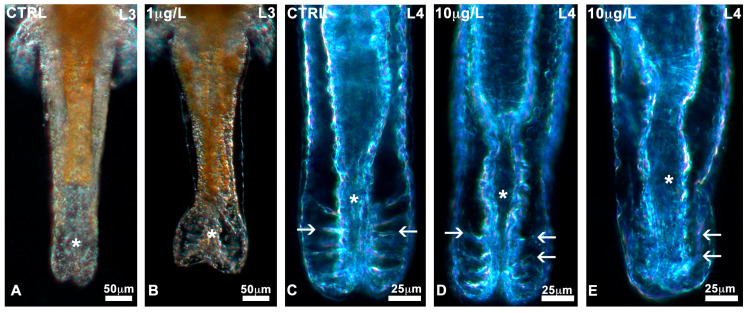
Alterations in the posterior abdomen of *Artemia salina* nauplii exposed to DLZ. (**A**) Normal condition (*). (**B**) Marked dilatation (*). Notice the different distributions of the yellow fat reserve. (**C**) Posterior gut (*) with normal ligaments (arrows). (**D**,**E**) Altered ligaments (arrows), posterior gut (*). Fixation in 4% formalin, no staining, in toto observation under incident light at different angles. Bars: (**A**,**B**), 50 µm; (**C**–**E**), 25 µm.

No significant alterations in the general body plan or appendages (Figure S1) were noticed during development in control nauplii or nauplii exposed to the three different concentrations of DLZ. All nauplii at stages L0, L1, and L2 had short, non-differentiated bodies containing a recognisable, dense, and yellowish gut. The head was characterised by a median naupliar eye and two pairs of antennae. At stage L3, the body was elongated, less dense, and eventually showed the first traces of thoracic segmentation. The development of thoracopod buds identified the L4 stage. These initially appeared as flat, short lateral protrusions that progressively lengthened and specialised. In DLZ-treated nauplii, alterations were observed at the level of the posterior region of the abdomen. No matter the concentration of DLZ, in about 0.5% of nauplii, it was dilated (Figure 3B) compared to the controls (Figure 3A). In addition, in about 30% of nauplii, the ligaments (Figure 3C) appeared poorly organised (Figure 3D) or moderately disorganised (Figure 3E).

### 3.4. Effects of DLZ on Eye Anatomy

**Figure 4:**
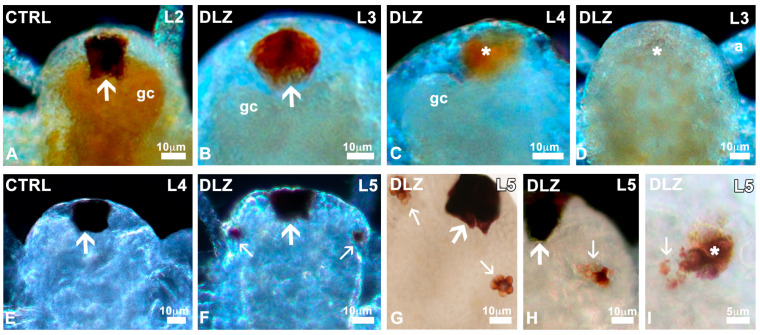
Eyes in *Artemia salina* nauplii exposed to DLZ. (**A**) Normal median eye (arrow) showing intense pigmentation. (**B**) Eye with irregular pigmentation (arrow). (**C**,**D**) Partially and completely depigmented eyes (*). Gastric caeca (gc), first antenna (a). (**E**,**F**) Appearance of the first paired eye buds (small arrows) laterally to the median eye (arrow). (**G**) Detail of the two buds (small arrows). (**H**) Irregular distribution of pigmentation (small arrow). Median eye (arrow). (**I**) Pigment (small arrow) outside the eye bud (*). Fixation in 4% formalin, no staining, in toto observation under transmitted (**G**–**I**) or incident light at different angles (**A**–**F**). Bars: (**A**–**H**), 10 µm; (**I**), 5 µm.

Nauplii showed a large and densely pigmented frontal eye since stage L1 (Figure 4A). In DLZ-treated nauplii, no matter the concentration or the stage of development, partial (Figure 4B,C) or total (Figure 4D) depigmentation was only occasionally recorded (4% of nauplii vs. 1.8% of controls).

In controls and treated nauplii, paired eyes, absent in stage L4 (Figure 4E), began to develop at the L4/L5 transition stage (Figure 4F). In controls and DLZ-treated nauplii, the two buds initially showed a different pigmentation (Figure S5) but rapidly developed, becoming well pigmented, appearing as a blackberry in both controls and most DLZ-treated nauplii (Figure 4G). In the latter group, however, no matter the concentration, abnormal paired eyes were occasionally (<5% of nauplii) observed; cells were irregularly pigmented (Figure 4H), or pigment appeared dispersed outside the eye contour (Figure 4I).

### 3.5. Effects of DLZ on Lipid Distribution

**Figure 5:**
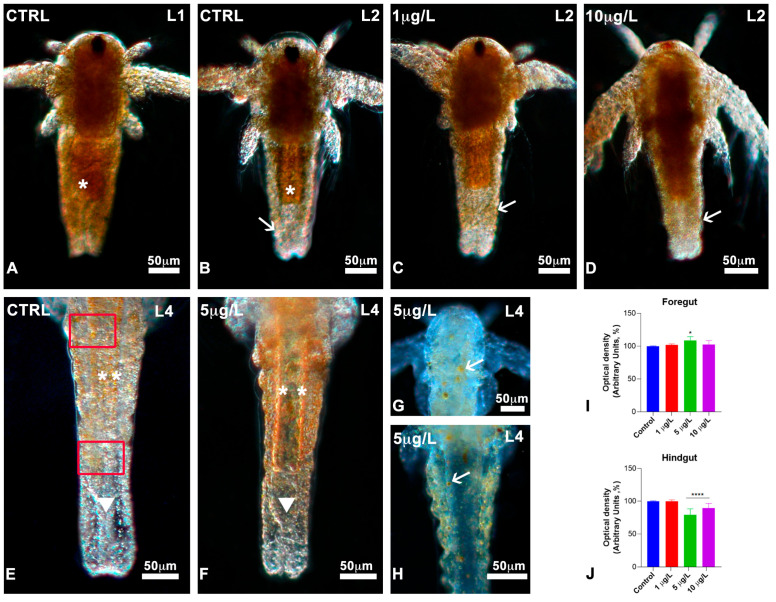
Lipid distribution during development in *Artemia salina* nauplii exposed to DLZ. (**A**) Dense, fat body (*). (**B**) Fat (*) decreases in the posterior abdomen and the hindgut (arrow). (**C**,**D**) Dense posterior abdomen (arrows). (**E**) Detail of the posterior abdomen; notice the dispersed yolk globules (**); hindgut region (arrowhead) completely devoid. In red, the areas in which absorbance was measured. (**F**) Detail of the posterior abdomen; presence of many yolk globules in the fore- (**) and hindgut (arrowhead) areas. (**G**,**H**) Presence of few, large yolk globules (arrows). Fixation in 4% formalin, no staining, in toto observation under incident light at different angles. Bars: 50 µm. (**I**,**J**) Optical density (grey values) measured in the areas indicated in red, in (**E**). Greyscale values: 0 = black; 256 = white. Two-Way ANOVA followed by Tukey’s pairwise comparison test; * *p* < 0.05; **** *p* < 0.0001. Number of animals examined: (**I**,**J**), n = 40 nauplii/treatment/gut area.

In stage L0 and L1 nauplii, no differences were noticed between controls and treatments: all nauplii showed a dense, fat body (Figure 5A), yellowish due to yolk globules. From the L2 stage, in controls, the fat began to disappear starting from the posterior abdomen and hindgut (Figure 5B). In L2 nauplii treated with DLZ, the disappearance was much less evident: the posterior area of the body remained opaque, and dense, at all concentrations (Figure 5C,D). At the end of the L3 stage, when thoracopod buds first appeared, in the controls, the fat globules were less numerous and more clearly recognisable since the body was almost entirely transparent (Figure 5E). In nauplii exposed to DLZ, about 30% of nauplii showed a significant persistence of fat (Figure 5F).

The data were confirmed by the absorbance analyses: values in the anterior portion of the abdomen did not differ significantly (Figure 5I), while in the posterior portion (Figure 5J), the DLZ-treated nauplii were substantially denser. The maximum effect size, 3.2, was registered for nauplii exposed to DLZ 5 µg/L; for 10 µg/L, the effect size was <0.5. Nauplii with large, residual fat globules were observed occasionally (Figure 5G,H).

### 3.6. Effects of DLZ on Heartbeat Rate

**Figure 6:**
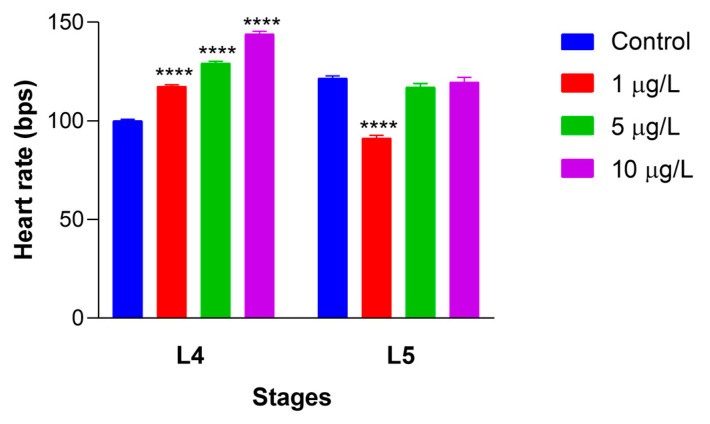
Alterations in heartbeat rate in *Artemia salina* nauplii exposed to DLZ. Two-Way ANOVA followed by Tukey’s pairwise comparison test; **** *p* < 0.0001. Number of animals examined: n = 40 nauplii/treatment/stage. Beats per second (bps).

The mean heart rate measured in control L4 and L5 (Figure 6) nauplii was 100 ± 4.4 and 121 ± 6.9 beats per minute. In L4 nauplii exposed to DLZ, the beat number rose dose-dependently, from 117.6 ± 4.7 in 1 μg/L to 129.2 ± 4.0 and 144.1 ± 5.7 in 5 and 10 μg/L, respectively.

In L5-treated nauplii, the beat number showed a significant decrease to 91.3 ± 8.4 at 1 μg/L and remained unchanged at 5 and 10 μg/L (117.1 ± 12.1 and 119.7 ± 16.5, respectively). The average effect size for L4 nauplii ranged from a minimum of 2.2 at 1 μg/L to a maximum of 9.3 at 10 μg/L. For L5 nauplii, the only significant effect size, 3.6, was registered for 1 μg/L treated nauplii.

### 3.7. Locomotor Performance of Nauplii

**Figure 7:**
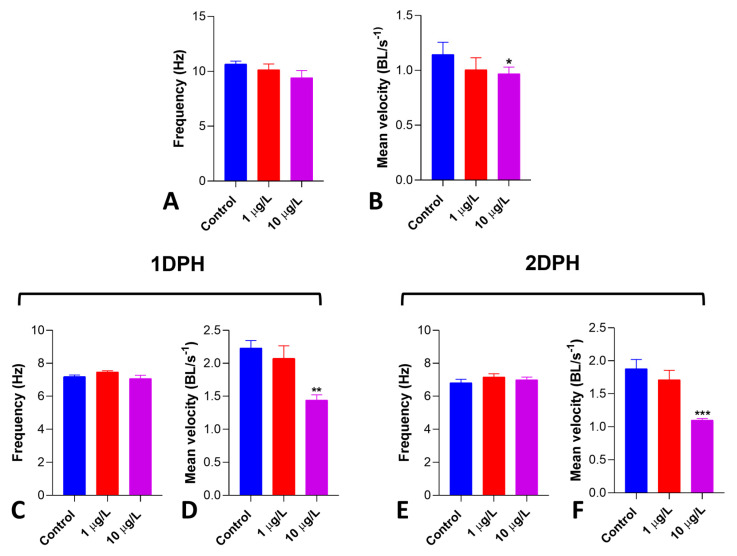
Effects of DLZ 1 and 10 µg/L on *Artemia salina* nauplii locomotor performance. Pre-hatching treatment. (**A**) Antennal stroke frequency (Hz) shows a noticeable, albeit not significant, decrease (*p* = 0.1941). (**B**) Mean velocity (BL/s) significantly decreases in 10 µg/L treated nauplii. Treatment started 1 or 2 days post-hatching. (**C**,**D**) No changes in antennal stroke frequency (Hz). (**E**,**F**) Mean velocity (BL/s) significantly decreases in 10 µg/L treated nauplii. Results are reported as means ± SD of values obtained after 48 h treatment. * *p* < 0.05; ** *p* < 0.01; *** *p* < 0.001. Number of animals examined: n = 10 nauplii/treatment.

In 2 dph nauplii obtained from cysts exposed at hydration (pre-hatching), DLZ had no effects on the antennal stroke frequency (Figure 7A). At the same time, it significantly reduced the mean velocity by about 35% at 10 µg/L (Figure 7B). In nauplii treated with DLZ at 1 (Figure 7C,D) or 2 days post-hatching (Figure 7E,F), similar results were obtained. No effects were registered on antennal stroke frequency (Figure 7C,E), while a significant reduction in mean velocity was observed at 10 µg/L (35% and 42% on 1 dph and 2 dph nauplii, respectively) (Figure 7D,F).

## 4. Discussion

Results indicate that DLZ can penetrate the cyst shell, an unperforated, semipermeable structure [44,45,46], interfering with *Artemia*’s early development. Mortality doubles, growth slows down, and moderate anatomical and functional damage occurs.

The first evidence of interference comes from the dose-dependent acceleration of hatching. The simplest explanation is that the DLZ accelerated embryo development, particularly cell proliferation, by promoting cell growth and DNA synthesis [47]. The effect would probably have been induced via peripheral benzodiazepine receptors, as already demonstrated in tumour cells, another fast-proliferating system [48]. It is also possible that DLZ, accumulating in the chitinous layer, favoured hatching by reducing cyst wall resistance [49]. No data are available for *Artemia*; however, forensic entomology proves that BZDs accumulate in the chitinous exuviae of insects [50,51], and we previously demonstrated that DLZ alters carbohydrate composition in *Mytilus* gill lamellae’s chitinous septa [20].

It cannot be excluded that DLZ interfered directly with the hatching process by altering the release of naupliar metabolites and/or secretions [52,53]. These compounds induce osmotic changes that lead to cyst water ingression, increase internal pressure, and eventually favour cyst wall breaking. In our experiments, the effect could have been exerted by altering gene expression via interference with genomic DNA methylation and/or promoters of developmental genes, an interference already proven in *Xenopus* embryos [19,54].

A further possible explanation of the anticipated hatching comes from the observation that DLZ, although anxiolytic and sedative, induces hyperactivity in the snail *Planorbarius corneus* [22]. The hypothesis that in *Artemia*, DLZ stimulated hatching by increasing antennal activity [52] is contradicted by behavioural results: DLZ reduces, not increases, the thrust generated by the antennae in neonate nauplii, as indicated by the reduced velocity.

Reduced naupliar size at hatching indicates that DLZ negatively interferes with early growth, and this would account for the observed increase in mortality. This conclusion seems in line with previous observations carried out in phylogenetically distant species: anticipated hatching correlates with reduced animal sizes [55] and is linked to a series of negative relapses in post-hatching growth and physiology [56]. According to Trabelsi et al. [55], the early-hatched larvae, though smaller, grow faster. In *Artemia*, the opposite occurs, and DLZ-exposed nauplii tend to be delayed, reaching less advanced stages than controls. Intriguing, however, is the evidence that the delay is reduced at a higher concentration of DLZ. Unfortunately, no explanation is currently possible since the mechanisms underlying the correlation between times of birth, growth, and health are not fully clarified.

The observed delayed development correlates well with the persistence of lipid reserve in L3/L4 nauplii. BZDs are lipophilic [57,58]; therefore, it can be supposed that DLZ dissolved in the fats reserve, which is abundant in *Artemia* embryos [59]. Based on what was reported in other vertebrate and invertebrate species, it can be hypothesised that, once internalised, DLZ might have interfered with metabolism [60], causing lipid peroxidation [61] and/or alterations in the expression of the enzymes involved in reserve resorption [25]. All these effects can be generated by DLZ binding to the mitochondrial peripheral TSPO receptors [62,63]. Altered mitochondria have been observed in *Xenopus* embryos exposed to the same concentrations of DLZ used in tests with *Artemia* [21].

Alteration in lipid reserve consumption may have impacted naupliar energetics, which would account for delayed development and reduced locomotor speed. To what extent this last effect depended on damage at the muscle level [21] remains to be evaluated.

Another point deserving of future attention is the supposed sedative effect of DLZ [62,64]. In *Artemia*, it appears improbable since the antennal stroke frequency showed no significant decrease. In *Xenopus* embryos [19], *Danio* larvae [65] and adult *Mytilus* [20], it induces the expected relaxation [66], but in the freshwater snail *Planorbarius*, the same concentrations cause locomotor hyperactivity [22]. In *Artemia*, sedation is also contradicted by the dose-dependent increased heartbeat rate, a proxy for stressful conditions in response to environmental variables [67]. The effect is not stage-dependent since frequency increases concomitantly with growth [68,69]. Tachycardia after exposure to BZDs is reported in mammals [70] and in in vitro cultured cardiomyocytes [71].

In recent papers, we demonstrated that DLZ is teratogenic for *Xenopus* embryos [19,54]. The observations carried out in *Artemia* suggest that DLZ is not teratogenic for this species: the body axes, the thoracopod buds, and the gut, visible from the external in transparent nauplii, appeared normally developed. BZDs, therefore, as with the other toxicants, exert specie-specific toxic effects [72,73]. In *Artemia*, the chitinous cyst wall and larval cuticle can only partially defend against DLZ [74]; a major defence is probably derived from the physiological adaptations to the extreme conditions this species has developed to survive in salty ponds [75].

Though not teratogenic, minor effects were noticed at the level of the eyes, which showed irregular pigmentation. Comparable alterations have already been observed in nauplii exposed to heavy metals [39], indicating that these structures are a target of environmental toxicity in this species, as in vertebrates [19,76,77]. No information on the compound eye development in *Artemia* was found. However, reports in other crustaceans indicate that several hundred genes are involved, with high similarities with *Drosophila* [78]. In addition, we demonstrated that DLZ can interfere with the genome [54] and eye development [21] in *Xenopus laevis*. Consequently, it is reasonable to hypothesise that the observed effects on compound eyes of *Artemia* depend on alterations in gene expression. Investigations to clarify this point are ongoing.

In *Xenopus*, significant alterations are induced by DLZ at the level of the skeletal muscles [21]. In *Artemia*, no data were collected on this aspect, but a dose-dependent decrease in mean swimming velocity was observed. Dorsal and ventral extrinsic limb muscles, well developed in this species since hatching [79], contain GABA receptors, as demonstrated in other arthropods [80] and crustaceans [81,82]. Occurrence, distribution, and properties in *Artemia salina* remain to be clarified before fully explaining benzodiazepine interference in this species’ development.

Results reported here come from a first series of investigations and require further verification, also considering that the literature on BZD toxicity in animal models, especially invertebrates, is scanty, and much information is still missing. Future investigations with multi-disciplinary approaches should first clarify DLZ toxicokinetics: knowing the half-life in water and tissues or the number and type of metabolites produced is essential. Cytological, ultrastructural, and molecular investigations, some of which are already in progress, will shed light on the mechanism of action of DLZ in a non-conventional target organism. Interferences with yolk and eyes were already reported in *X. laevis* [19,21], but parallels between evolutionarily distant species such as an amphibian and a crustacean require careful confirmation.

## 5. Conclusions

The preliminary evidence indicates that DLZ negatively affects early development in *Artemia salina* nauplii, confirming the necessity of increasing attention to the environmental risks associated with improperly discarding pharmaceuticals. Knowing such risks could lead to more careful and timely information to citizens and push toward a more conscious and respectful use of BZDs.

Future cytological, biochemical, and molecular investigations will clarify DLZ’s mechanism of action on this species, making it a model organism for toxicity testing and a reference for predicting the health status of marine communities. Crustacean nauplii represent a relevant component of zooplankton, which is, in turn, at the base of the trophic food chain. Therefore, any disturbance at this level could have severe repercussions on higher levels, threatening the stability of the entire aquatic environment.

## Data Availability

Data are contained within the article and Supplementary Materials.

## Supplementary Materials

The following supporting information can be downloaded at: https://www.mdpi.com/article/10.3390/biology13100808/s1, Figure S1: Naupliar anatomy during early development. (A) Prenauplius in the naupliar membrane (arrowhead). (B) Free swimming nauplius with dense body wall (*). (C) Transparent body wall with evidence of segmentation (dotted arrows). (D) Thoracopod buds (dotted arrow). (E) Long, undifferentiated thoracopods (t). Appearance of the paired eye (small arrow). (F) Long thoracopods (t) and evident paired eye (small arrow). Gut (**), naupliar eye (arrows). Fixation in 4% formalin, no staining, in toto observation under incident light. Bars: 50 µm. Table S1: Groups of Artemia salina nauplii used for protocols A and B, as described in the main text. For each group, 5 batches of cysts were incubated. Days post hydration (dph). Figure S2: (A) The arena used to evaluate the Artemia nauplii’s locomotory performance. The arena was square, 1 mm deep, with a transparent bottom, back-illuminated, and observed under a stereomicroscope. (B) Types of locomotory activity observed in the arena. Figure S3: (A) Example of ethogram showing the time spent by a control nauplius in one of the three swimming activities described in Figure 1 over a 20 s period. (B) Time spent (in %) by Artemia nauplii of 1 to 4 dph while circular swimming, thigmotactic swimming, and straight swimming. Two-Way ANOVA followed by Tukey’s multiple comparison tests demonstrated that the animals spend most of their time in straight swimming (*p* < 0.05), and there was no significant change with age. Figure S4: Oscillations of instantaneous velocity during jump-swimming of Artemia nauplii. The dashed line represents the mean velocity. Figure S5: Different pigmentation of the two buds (arrow and dotted arrow) of the paired eye in Artemia salina nauplii (stage L4/L5). No differences were detected among controls (A) and DLZ-treated (B) nauplii. N = naupliar eye; a = antennae. See ref. [83].
