# Supplementary material for: Impact of Benzodiazepine Delorazepam on Growth and Behaviour of Artemia salina Nauplii
Source: Biology (Basel). 2024 Oct 10;13(10):808. doi: 10.3390/biology13100808 (PMC11505015; doi:10.3390/biology13100808)
Supplement: Supplementary file 1 [file biology-13-00808-s001.zip › biology-3195866-supplementary.pdf]

Supplementary Material

Impact of Benzodiazepine Delorazepam on Growth and Behavior of *Artemia salina* nauplii

Chiara Fogliano[1], Rosa Carotenuto[1], Claudio Agnisola[1], Chiara Maria Motta[1*] and Bice Avallone[1]

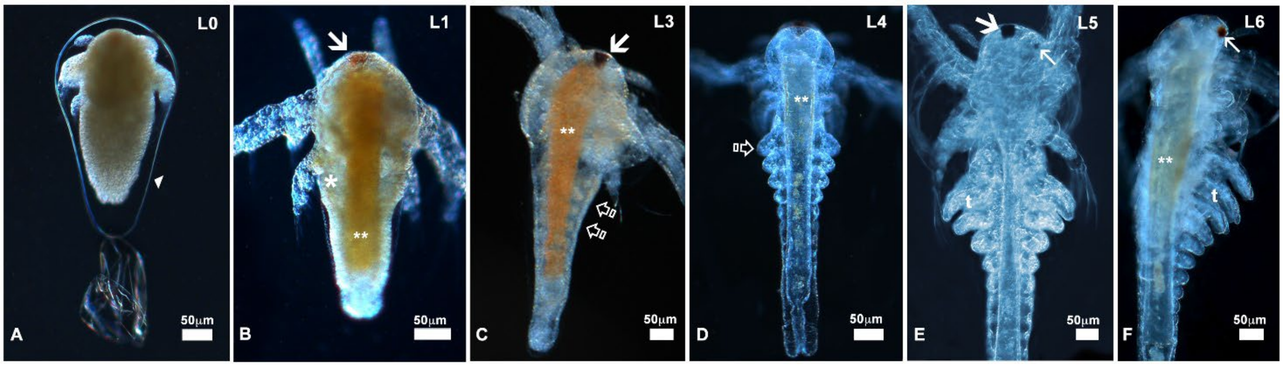

Figure S1. Naupliar anatomy during early development. A) Prenauplius in the naupliar membrane (arrowhead). B) Free swimming nauplius with dense body wall (*). C) Transparent body wall with evidence of segmentation (dotted arrows). D) Thoracopod buds (dotted arrow). E) Long, undifferentiated thoracopods (t). Appearance of the paired eye (small arrow). F) Long thoracopods (t) and evident paired eye (small arrow). Gut (**), naupliar eye (arrows). Fixation in 4% formalin, no staining, *in toto* observation under incident light. Bars: 50 µm.

Evaluation of locomotory activity in nauplii of *Artemia salina*

A continuous swimming activity characterises *Artemia salina*; unlike the adults, which display metachronal swimming, the *Artemia salina* nauplii swim with one pair of limbs, the second antennae, that dominate propulsion during the first half of larval life, resulting in jerky swimming (jump-swimming, mainly studied in copepod nauplii, [1]). We evaluated the locomotory performance of *Artemia* nauplii by using a 1 cm squared arena (Fig. S2A), where the nauplii were tested individually. In a preliminary behavioral study, we observed three types of nauplii swimming behavior (Fig. S2B): circular, thigmotactic, and straight. The preliminary analysis also demonstrated that nauplii spent 60 to 67 % of the time straight swimming (Fig. S3).

We analysed locomotory performance only during straight swimming, which was the one with the lower statistical variability and results from the symmetrical working of the antennae. In particular, we determined the individual mean speed (cm/s), and the number of antennae beats (Hz) from two replicate video recordings (1920 x 1080 px, 30 fps, 1 s duration) using the Tracker software (Tracker 6.1.7, 2023, OSP). Position and instantaneous velocity were determined at 0.03 s intervals. Fig. S4 reports an example of the time record of instantaneous velocity in a 1dph nauplius. According to the jump-swimming modality, instantaneous velocity oscillates between a positive trust during

the backward movement of the antennae and a negative trust during the forward movement of the antennae. The mean velocity is positive and related to the antennae's muscular contraction strength. The number of beats was calculated as the number of velocity peaks per second

Table S1. Groups of *Artemia salina* nauplii used for protocols A and B, as described in the main text. For each group, 5 batches of cysts were incubated**.** Days post hydration (dph).

		dph				
		0	1	2	3	4
Protocol A (pre-hatching)				Control<br>Treated 1 μg/L<br>Treated 10 μg/L		
Protocol B	B1: No treatment				Control B1	
	B2: No treatment					Control B2
	B1: treatment at 1 dph				Treated 1 μg/L<br>Treated 10 μg /L	
	B2: treatment at 2 dph					Treated 1μg/L<br>Treated 10 μg/L

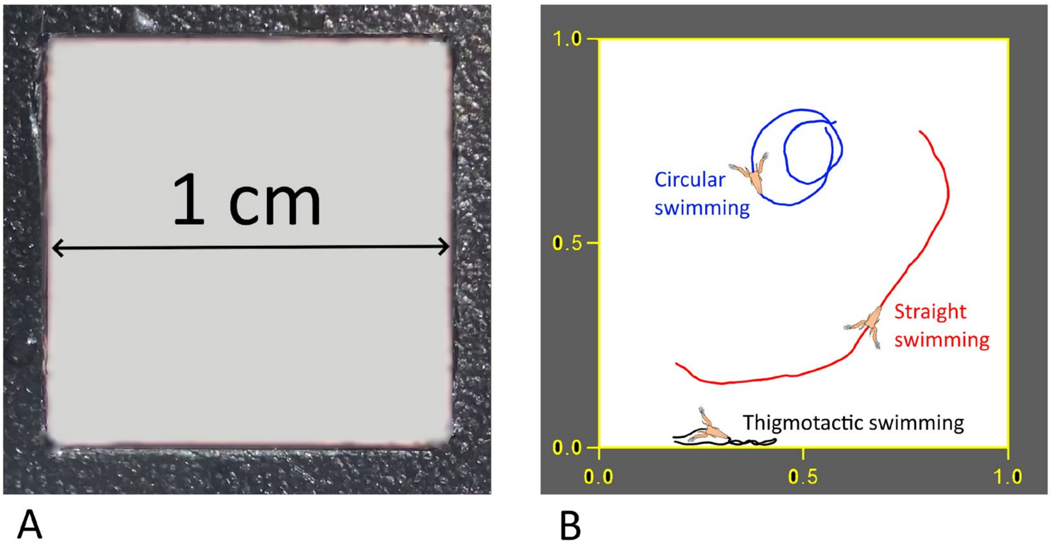

Figure S2. A) The arena used to evaluate the *Artemia* nauplii's locomotory performance. The arena was square, 1 mm deep, with a transparent bottom, back-illuminated, and observed under a stereomicroscope. B) Types of locomotory activity observed in the arena.

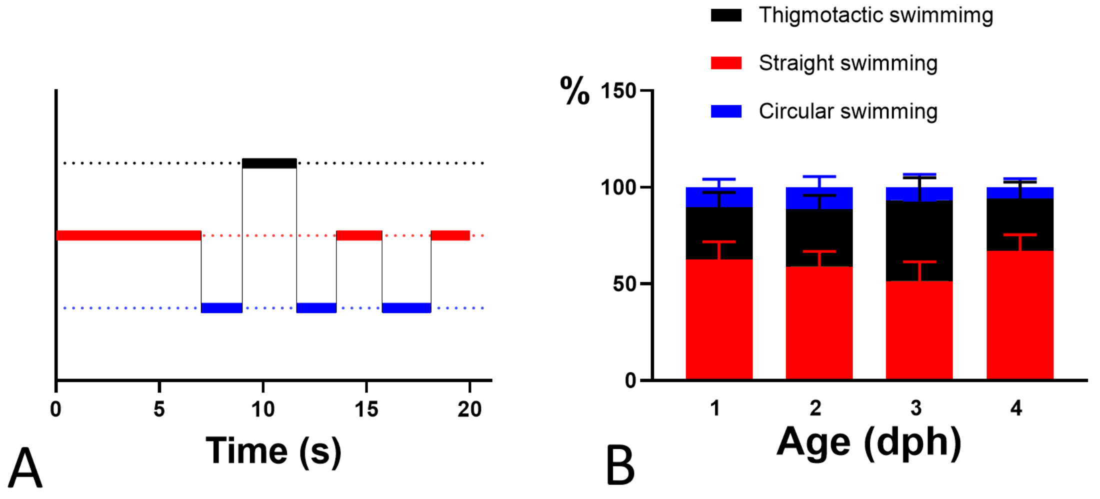

Figure S3. A) Example of ethogram showing the time spent by a control nauplius in one of the three swimming activities described in Fig. 1 over a 20 s period. B) Time spent (in %) by *Artemia* nauplii of 1 to 4 dph while circular swimming, thigmotactic swimming, and straight swimming. Two-Way ANOVA followed by Tukey's multiple comparison tests demonstrated that the animals spend most of their time in straight swimming ($p<0.05$), and there was no significant change with age.

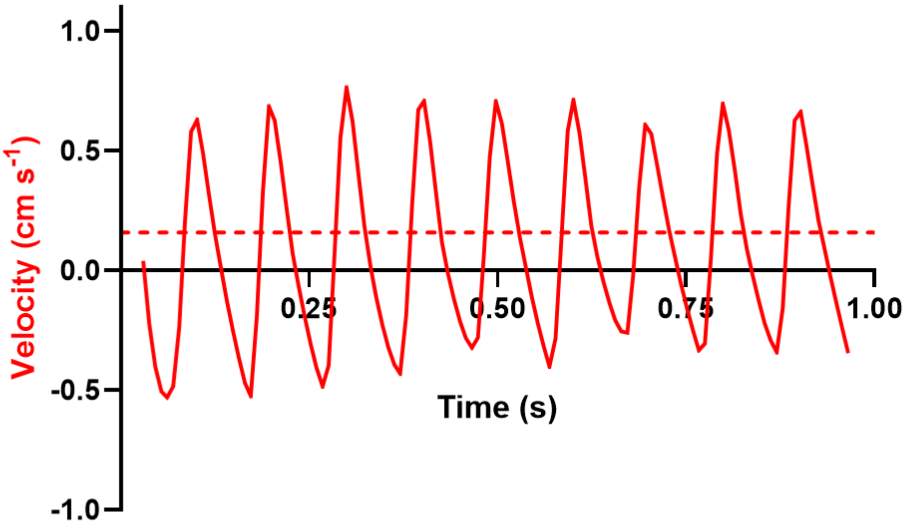

Figure S4. Oscillations of instantaneous velocity during jump-swimming of *Artemia* nauplii. The dashed line represents the mean velocity.

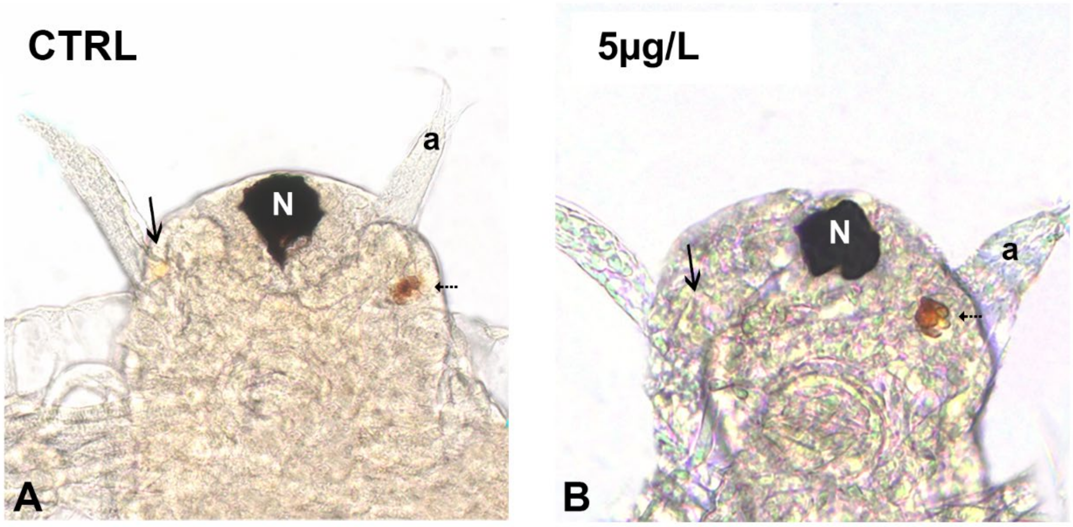

Figure S5. Different pigmentation of the two buds (arrow and dotted arrow) of the paired eye in *Artemia salina* nauplii (stage L4/L5). No differences were detected among controls (A) and DLZ-treated (B) nauplii. N= naupliar eye; a= antennae.

References

[1] Wadhwa, N., Andersen, A., Kiørboe, T. (2014). Hydrodynamics and energetics of jumping copepod nauplii and copepodits. Journal of Experimental Biology, 217(17), 3085-3094. https://doi.org/10.1242/jeb.105676
